# Lupeol restores dopaminergic function by suppressing glial activation in a Parkinson’s disease mouse model

**DOI:** 10.3389/fimmu.2026.1708581

**Published:** 2026-02-02

**Authors:** Riaz Ahmad, Kyonghwan Choe, Hyun Young Park, Waqas Ahmad, Tae Ju Park, Myeong Ok Kim

**Affiliations:** 1 Division of Life Sciences and Applied Life Science (BK 21 Four), College of Natural Science, Gyeongsang National University, Jinju, Republic of Korea; 2 Department of Psychiatry and Neuropsychology, School for Mental Health and Neuroscience (MHeNs), Maastricht University, Maastricht, Netherlands; 3 Department of Pediatrics, Maastricht University Medical Center (MUMC+), Maastricht, Netherlands; 4 Department of Cell Biology, Albert Einstein College of Medicine, Bronx, NY, United States; 5 Alz-Dementia Korea Co., Jinju, Republic of Korea

**Keywords:** microglial activation, motor dysfunction, neuroinflammation, oxidative stress, Parkinson’s disease (PD)

## Abstract

**Introduction:**

Parkinson’s disease (PD) is a progressive neurodegenerative disorder marked by chronic neuroinflammation and loss of dopaminergic neurons. The neurotoxin MPTP (1-methyl-4-phenyl-1,2,3,6-tetrahydropyridine) selectively targets dopaminergic neurons, effectively replicating the pathological features of PD. Lupeol, a natural pentacyclic triterpenoid, has been shown to exhibit neuroprotective properties in various models by reducing oxidative stress, inflammation, and apoptosis. This study aimed to investigate the neuroprotective effects of lupeol in an MPTP-induced mouse model of PD.

**Methods:**

Male mice were administered MPTP (30 mg/kg, i.p.) for seven days to induce PD-like pathology. Lupeol (50 mg/kg) was administered as a potential therapeutic intervention. Behavioral assessments were conducted to evaluate motor function. Biochemical analyses were performed to measure dopamine and tyrosine hydroxylase (TH) levels. Immunohistochemical and molecular approaches were used to assess glial activation, oxidative stress, and apoptotic signaling pathways in the substantia nigra pars compacta (SNpc) and striatum.

**Results:**

Lupeol treatment significantly improved MPTP-induced motor impairments and restored dopamine and TH levels. Additionally, lupeol reduced neuroinflammation, by decreasing microglial activation, astrocyte reactivity, and lower levels of inflammatory mediators. Oxidative stress markers, including reactive oxygen species (ROS) and lipid peroxidation (LPO), were diminished in SNpc and striatum following lupeol treatment. Furthermore, lupeol upregulated antioxidant defense mechanisms by increasing the expression of Nrf-2 and HO-1. Apoptotic markers, such as Cytochrome C, Bax, and Caspase-3, were downregulated, indicating reduced neuronal apoptosis.

**Conclusion:**

The current study suggests that lupeol exerts neuroprotective effects by inhibiting glial cell activation, thereby reducing neuroinflammation, oxidative stress, and apoptosis in an MPTP-induced PD mouse model.

## 1 Introduction

Parkinson’s disease (PD), the second most predominant neurodegenerative disorder mostly affecting older adults (1), is considered by the progressive degradation of dopamine (DA) neurons in the striatal dopaminergic nerve terminals and substantia nigra pars compacta (SNpc), which are abundant in DA transporters (2, 3). Dopamine release via the nigrostriatal pathway modulates corticostriatal transmission in medium-spiny neurons which express dopamine receptors, leading to either motor stimulation or inhibition, respectively (4). In PD, the loss of DA neurons in the SNpc is thought to induce extensive motor inhibition, with diverse effects on neurons expressing D1 and D2 receptors (5–7). Although the actual etiology of PD is still to be fully established, various system-level mechanisms and dysfunctions, including mitochondrial dysfunctions, dopamine homeostasis, neuroinflammation, and autophagy, have been associated with the development of PD (8).

Most assumptions concerning the cause and development of dopamine insufficiency in PD have been formulated based on studies conducted in animal models. The 1-methyl-4-phenyl-1,2,3,6-tetrahydropyridine (MPTP) model is one of the most frequently used in preclinical PD studies. MPTP administration consistently causes neurotoxic lesions in the nigrostriatal dopaminergic system, with persistent and reproducible findings (9). MPTP demonstrates neurotoxicity, causing a cascade of negative reactions including inflammation, oxidative stress, mitochondrial apoptosis, excitotoxicity, and inclusion formation. These pathways, both independently and synergistically, cause dopaminergic neuron destruction in the SNpc and striatum (10) and an increase in astrocyte and microglial activation (11). MPTP can penetrate the blood-brain barrier and destroy dopaminergic neurons of the nigrostriatal pathway (12). The MPTP protoxin is metabolized in non-dopaminergic cells in the brain by the enzyme monoamine oxidase B (MAO-B) to 1-methyl-4-phenyl-2,3-dihydropyridinium (MPDP+) and then to 1-methyl-4-phenylpyridinium (MPP+). MPP+ triggers an inflammatory response characterized by T cell infiltration into the striatum and SNpc, accompanying the activation of microglia and astrocytes (11, 13, 14), leading to the secretion of pro-inflammatory cytokines such as TNF-α and IL-1β, which have detrimental effects on dopamine neurons in animal models of PD (15–17). Furthermore, MPP+ is released into the extracellular space, where its polarity precludes it from freely entering cells. Instead, it depends on plasma membrane transporters to obtain access to dopaminergic neurons (18, 19). This is why neurons and other catecholaminergic neurons are particularly vulnerable to MPTP-induced degeneration. This sensitivity is caused by the selective absorption of MPP+ by the dopamine transporter (20, 21), where it binds to the vesicular monoamine transporter (VMAT) and subsequently accumulates in the cytoplasm where it can interact with numerous enzymes and potentially localize within the mitochondria (7, 22). Within the mitochondria, MPP+ impedes complex I of the electron transport chain, leading to impaired ATP production and increased generation of reactive oxygen species (23–26), that lead to oxidative stress, and further induce the release of cytochrome c from the mitochondria into the cytosol, triggering the caspase activation cascade (27), which upregulates proapoptotic proteins (28, 29) and ultimately results in dopaminergic cell death.

Natural products are promising candidates for developing novel therapeutic interventions for PD owing to their diverse bioactive qualities (30, 31). Levodopa, dopamine agonists, MAO-B inhibitors, COMT inhibitors, and anticholinergic drugs are among the approved treatments for Parkinson’s disease (PD) (32). Despite the fact that these medications successfully alleviate motor symptoms, they only provide symptomatic relief and have a limited overall benefit due to long-term side effects include dyskinesia, motor fluctuations, and cognitive adverse effects. Crucially, none of the existing therapies stop neurodegeneration. These drawbacks emphasize the need for new drugs that have both neuroprotective and disease-modifying properties. The antioxidant, anti-inflammatory, and anti-apoptotic qualities of natural substances like lupeol are being investigated more and more (33). Lupeol, a triterpenoid compound, ubiquitously exists in various vegetables and fruits including mango, strawberry, olive, red grapes, cucumber, white cabbage, tomato, and green pepper, as well as in traditional medicinal herbs (34, 35). From a pharmacological perspective, lupeol demonstrates multiple therapeutic functions including anti-inflammatory, antioxidant, anti-cancer, and anti-apoptotic functions, which are effective against various diseases (34, 36). Additionally, lupeol exerts neuroprotective effects via suppressing microglial activation and may alleviate mitochondrial dysfunction, thereby reducing ATP depletion, oxidative stress, and apoptosis (37–39). Nevertheless, it remains to be determined whether lupeol reduces dopaminergic neuron death by decreasing glial cell activation. In this study, we explored the potential of lupeol to inhibit MPTP-induced PD pathology by administering MPTP to adult male mice. After administering MPTP, we confirmed the successful induction of PD by observing behavioral impairments in the mice and by evaluating changes in the expression of TH+ fibers in the striatum and the SNpc. Our results imply that lupeol may play a vital and functional role in the pathophysiology of PD by regulating neuroinflammation and motor function, thereby presenting new therapeutic avenues for treating the disease.

## 2 Materials and methods

### 2.1 Lupeol preparation and administration

**Figure 1:**
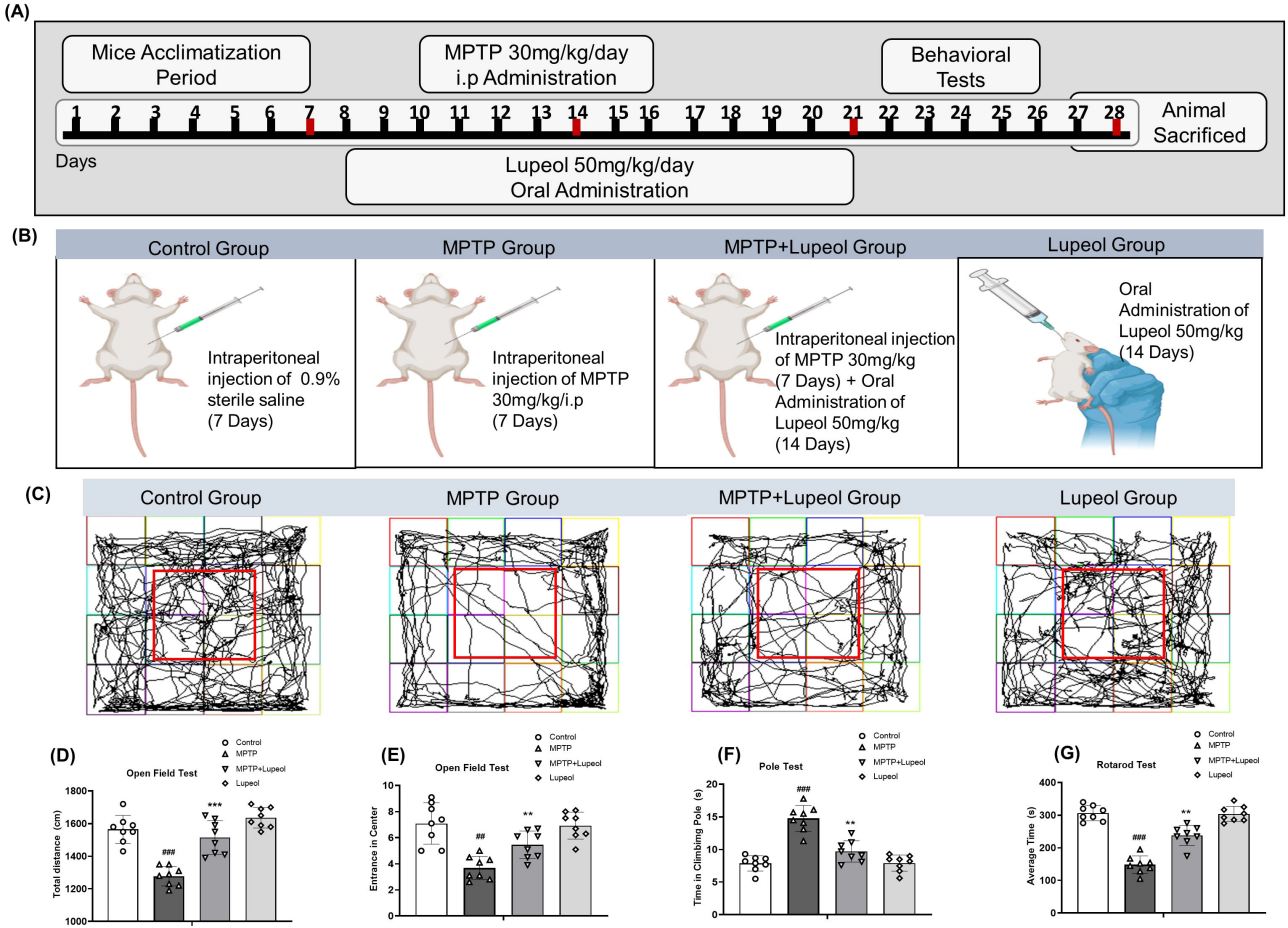
Lupeol mitigated MPTP-induced motor impairment in the Parkinson’s Disease (PD) mouse model. **(a)** Schematic representation of the experimental timeline for MPTP injections and lupeol administration. **(b)** Representative images of the experimental group of mice. **(c)** Illustrative images of open-field test apparatus. **(d, e)** Quantitative analysis of total distance traveled and time spent in the center region in the open-field test (n = 8/group). **(f)** Pole test assessing motor coordination. **(g)** Quantitative analysis of latency to fall in the rotarod performance test. Data are presented as mean ± SEM. The hash (#) sign indicates a significant difference from the control group; the sign asterisk (*) indicates a significant difference from the MPTP-treated group. Statistical significance: #p < 0.05; ##p < 0.01; ###p < 0.001, *p < 0.05; **p < 0.01; ***p < 0.001.

Lupeol (CAS Number: 545-47-1) was purchased from Sigma Co. (St. Louis, MO, USA). A stock solution was prepared by dissolving lupeol in dimethyl sulfoxide (DMSO). Each day, a fresh lupeol solution was prepared in normal saline according to the required injection volume. Mice were treated with lupeol at a dose of 50 mg/kg via oral administration. The dosage was given as per previous published study (40). The chemical structure of lupeol is shown in **Figure 1**.

### 2.2 Animals and group design

Eight-week-old male C57BL/6 mice weighing 25 and 30 grams were purchased from Samtako Bio (Osan, Republic of Korea). By using male mice, the confounding effects of hormone changes related to the estrous cycle are reduced. Progesterone and estrogen have neuroprotective properties that may change how well lupeol works. Each cage contained four mice under particular pathogen-free conditions. All mice were reared and kept on a 12-hour light/dark cycle with free access to food and water and were kept at a constant temperature and humidity. All the experimental animal techniques were authorized by the Animal Ethics Committee of the Division of Applied Life Sciences, Gyeongsang National University, South Korea (Approval ID: 125, Code: GNU-200331-M0020). The experimental approach is schematically represented in **Figure 1A**. Following a week of acclimation, mice were randomly divided to experimental groups using a computer-generated random sequence;

Control group *(n =* 08*)*: Treated with 0.9% sterile saline via intraperitoneal injection.

MPTP group *(n =* 08*)*: Treated with MPTP (30 mg/kg) followed by previous study (41), via intraperitoneal injection for 7 days.

MPTP + Lupeol group *(n =* 08*)*: Treated with MPTP (30 mg/kg) via intraperitoneal injection and Lupeol (50 mg/kg) via oral administration for 14 days.

Lupeol (sham) group *(n =* 08*)*: Treated only with Lupeol (50 mg/kg) via oral administration.

Every experiment was carried out in a blinded manner. The researchers conducting behavioral tests, tissue processing, imaging, and quantitative analysis were not informed of the group identities; instead, the animals were coded by a separate lab member. Anonymized codes were used to mark all histology slides, photos, and biochemical samples, and blinded datasets were used for statistical analysis. Only after all analyses were finished was group information disclosed.

### 2.3 Behavioral tests

#### 2.3.1 Open-field test

The open-field test is a useful technique in preclinical research for measuring motor impairments (42) and determining the efficacy of prospective therapies in PD mice models. We conducted this test to observe behavioral changes in mice, utilizing an enclosure with dimensions of 50 cm in length, 50 cm in breadth, and 40 cm in height, with white walls. Mice were placed singly in the center of the box, and for several minutes, they were allowed to acclimate to their new surroundings. Their movement was then captured with a camera for about 8 minutes. Behavioral parameters were quantified using the SMART video-tracking system (Panlab, Harvard Apparatus; Bioscience Company, Holliston, MA, USA) to confirm accurate and automated measurement of mice movements. After each trial, the box was cleaned with 70% alcohol and dried to eradicate any lingering odors.

#### 2.3.2 Pole test

The pole test is commonly used to evaluate motor coordination in PD mice (43). To keep the mice from slithering, a gauze bandage was enfolded around a metal rod with a diameter of 1 cm and a length of 50 cm, and the lower portion of the metal rod was inserted into the cage. The mice were positioned at the top of the rod, facing downward. The time it took for the mouse to return to the cage freely was recorded, peaking when its hind limbs struck the bottom. Mice were trained for two days before undergoing formal testing. Each mouse was evaluated three times, with a 15-minute delay between examinations, and the mean score was used for analysis.

#### 2.3.3 Rotarod test

The rotarod test was conducted to assess the motor function of the mice (44). They underwent training for 3 consecutive days before the drug administration. Following Lupeol and MPTP treatment, the mice underwent three trials, each with a 15-minute interval between them. During formal testing, each mouse was placed on a rotarod at speeds of 12 or 20 revolutions per minute (rpm). The duration of the mouse’s stays on the rotarod and the latency to fall was recorded and measured. Each trial’s latency to fall (time spent on the rod) was recorded using the Rotarod Analysis System.

### 2.4 Sample collection and tissue preparation

To collect fresh brain tissue, the mice were profoundly anesthetized with an intraperitoneal dose of pentobarbital sodium (50 mg/kg). The striatum and SN were promptly removed from the brain on ice and preserved at -80 °C. For histological study, mouse brain tissues were preserved overnight at 4 °C with 4% paraformaldehyde (PFA) in 0.01 M phosphate buffer (pH 7.4). The tissues were then moved to a 20% sucrose solution and stored at 4 °C overnight, followed by a 30% sucrose solution at 4 °C overnight for cryoprotection. The tissues were embedded in an optimal cutting temperature compound (OCT Compound, Tissue-Tek, USA) and cryosectioned at 10 μm with a cryostat microtome (CM1950, Leica, Germany).

### 2.5 Brain homogenization

To extract proteins, prepared brain tissue from the striatum and substantia nigra (SN) was homogenized and lysed in an ice-cold lysis solution containing protease and phosphatase inhibitors, as described before (45). Protein samples were obtained and quantified using a bicinchoninic acid kit (Sigma-Aldrich, St. Louis, MO, USA).

### 2.6 ROS assay

The ROS assay assessed the reactive oxygen species (ROS) levels in the brains of the experimental mice as described previously (46) (*n =* 08 mice/group). Briefly, brain homogenates from control, MPTP, and lupeol-treated groups were diluted 1:20 in ice-cold Lock’s buffer, resulting in a final concentration of 2.5 mg tissue/500 μL. The final reaction mixture, consisting of 1 mL of Lock’s buffer (pH ~7.4), 0.2 mL of homogenate, and 10 μL of DCFH-DA (5 mM), was incubated for 15 minutes at room temperature, allowing the conversion of DCFH-DA to the fluorescent component DCF. The conversion was assessed using a spectrofluorimeter with an excitation wavelength of 484 nm and an emission wavelength of 530 nm. Parallel blanks were used to measure background fluorescence in the absence of homogenate. ROS levels were quantified and expressed as relative pmol DCF/mg protein.

### 2.7 LPO assay

The lipid peroxidation (LPO) assay was conducted as earlier described, with some changes (47). Evaluating LPO is crucial for assessing oxidative stress. The LPO marker malondialdehyde (MDA) in SNpc and striatum protein lysates was measured using a thiobarbituric acid reactive substances (TBARS) assay kit (Bio Vision, Milpitas, CA, USA) following the manufacturer’s instructions.

### 2.8 Immunoblotting

Proteins were isolated using polyacrylamide gel electrophoresis with 10-12% gels, following previously described methods (45) with some modifications. Proteins were separated evenly using 10% sodium dodecyl sulfate-polyacrylamide gel electrophoresis (SDS-PAGE) and subsequently transferred onto polyvinylidene fluoride (PVDF) membranes (Millipore, Boston, MA, USA). To prevent nonspecific protein binding, the membranes were treated with 5% skim milk in Tris-buffered saline having Tween 20 (TBST) for one hour at room temperature. Following blocking with 5% skim milk for 1–2 h at room temperature, the PVDF membranes were treated with primary antibodies overnight at 4 °C. The membranes were then rinsed with TBST before being treated with the appropriate secondary antibodies for an hour at room temperature. The blots were visualized with an enhanced chemiluminescence system, and greyscale values were measured using ImageJ software (National Institutes of Health).

### 2.9 Immunofluorescence

Transcardial perfusion was performed with 1X phosphate-buffered saline (PBS) followed by 4% ice-cold paraformaldehyde as previously described (48). Brain tissues were fixed overnight in 4% paraformaldehyde before being transferred to 20% sucrose, and allowed to sink to the bottom of the tube. The brain was embedded in OCT (Tissue-Tek O.C.T. Composite Medium, Sakura Finetek USA, Inc., Torrance, CA, USA) and then sectioned into 14-μm coronal sections using a CM 3050S cryostat (Leica, Wetzlar, Germany). The sections were put on positively charged Probe-On slides (Thermo Fisher Scientific Inc., Waltham, MA, USA). Anatomically matched levels of the substantia nigra and striatum were used to obtain three coronal slices (thickness: 30 μm) for each brain. Using the same imaging conditions, three to five microscopic fields were taken from each slice. For quantitative analysis, nine to fifteen pictures of each animal were employed. To reduce variability, only portions with consistent anatomical landmarks were included, and all photos were obtained under blinded settings. For immunofluorescence staining, the slides were washed twice in 0.01 M PBS for 10 minutes each, then incubated for 1 hour in a blocking solution containing 2% normal serum (appropriate for the antibody used) and 0.3% Triton X-100 in PBS. After blocking, the slides were incubated overnight at 4 °C with primary antibodies (diluted 1:100 in blocking solution, (refer to **Table 1** for antibodies details). Following incubation with the primary antibody, the sections were then incubated with secondary antibodies labeled with tetramethyl rhodamine isothiocyanate (TRITC) or fluorescein isothiocyanate (FITC) (1:50; Santa Cruz Biotechnology, Dallas, TX, USA) for 2 hours. The slides were mounted using DAPI and Prolong Antifade Reagent (Molecular Probes, Eugene, OR, USA). The images of immunofluorescence staining were then analyzed using a confocal laser scanning microscope (Flouview FV 1000, Olympus, Japan).

**Table 1:** List of antibodies used in western blot and confocal microscopy.

Antibody	Host	Application	Manufacturer	Catalog number	Concentration
TH	Rabbit	WB/IF	Cell Signaling	E2L6M	1:1000/1:100
DAT	Rat	WB	Santa Cruz Biotechnology, United States	SC32259	1:1000
VMAT2	Mouse	WB	Santa Cruz Biotechnology, United States	SC374079	1:1000
Iba-1	Rabbit	WB/IF	Abcam	Ab178846	1:1000/1:100
GFAP	Mouse	WB/IF	Santa Cruz Biotechnology, United States	SC33673	1:1000/1:100
p-NF-кB	Mouse	WB	Santa Cruz Biotechnology, United States	SC136548	1:1000
TNF-α	Mouse	WB	Santa Cruz Biotechnology, United States	SC52746	1:1000
IL-1β	Mouse	WB	Santa Cruz Biotechnology, United States	SC32294	1:1000
COX2	Mouse	WB	Santa Cruz Biotechnology, United States	SC376861	1:1000
Nrf2	Mouse	WB/IF	Santa Cruz Biotechnology, United States	SC365949	1:1000/1:00
HO-1	Mouse	WB	Santa Cruz Biotechnology, United States	SC136961	1:1000
NeuN	Mouse	WB/IF	Cell Signaling	D4G4O	1:1000/1:00
Bax	Mouse	WB	Santa Cruz Biotechnology, United States	SC23959	1:1000
Bcl-2	Mouse	WB	Santa Cruz Biotechnology, United States	SC7382	1:1000
Cyto-C	Mouse	WB	Santa Cruz Biotechnology, United States	SC13156	1:1000
Casp-3	Rabbit	WB/IF	Cell Signaling	9662S	1:1000/1:00
β-actin	Mouse	WB	Santa Cruz Biotechnology, United States	SC47778	1:1000

### 2.10 Statistical analysis

The Western blot bands were densitometrically scanned and analyzed using a Sigma Gel computer system (SPSS Inc., Chicago, IL, USA). Immunofluorescence findings were analyzed with ImageJ software, and the densities were expressed in arbitrary units. Statistical analysis and graph generation were conducted using GraphPad Prism, Version 8.0 (GraphPad Software, San Diego, CA, USA). Data were given as mean ± SD and analyzed using one-way ANOVA with Tukey’s test for multiple comparisons. A P-value < 0.05 was considered a significant difference between groups.

## 3 Results

### 3.1 Lupeol treatment attenuated the motor dysfunction in MPTP-induced PD model mouse

The open-field test is a widely used behavioral model for assessing locomotor activity and emotional responses in rodents (49). In this study, MPTP administration significantly reduced line-crossing frequency and increased passive sitting duration compared to the control group. Lupeol treatment effectively mitigated MPTP-induced impairments in locomotor activity (**Figures 1C–E**). To assess MPTP-induced bradykinesia, the pole test was performed across all experimental groups. Mice in the MPTP group exhibited prolonged descent times, indicating motor dysfunction. Notably, lupeol-treated mice demonstrated improved performance in the pole test (**Figure 1F**). Additionally, the rotarod test was conducted to evaluate motor deficits in the PD model. MPTP-treated mice showed a significant reduction in rotarod performance time, reflecting impaired motor coordination. Lupeol treatment significantly prolonged fall latency compared to the MPTP group, suggesting an improvement in motor function (**Figure 1G**).

### 3.2 Lupeol administration prevents the loss of DA neurons in the SNpc and DA nerve fibers in the striatum

**Figure 2:**
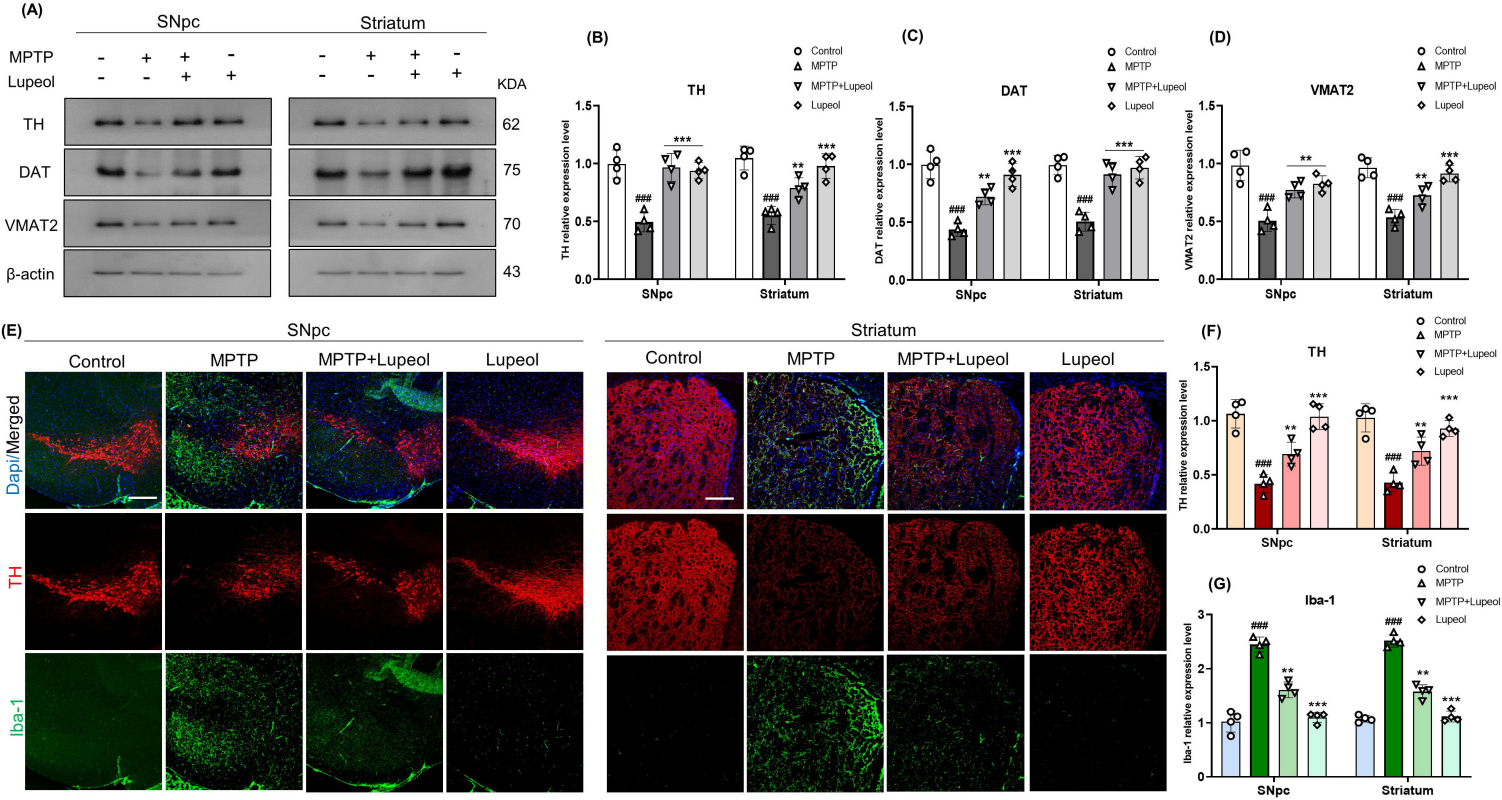
Lupeol averts the loss of Dopaminergic neuron (DA) neurons in the Substantia nigra pars compacta (SNpc) and DA nerve fibers in the striatum. **(a)** Western blot analysis for detecting the protein level of Tyrosine hydroxylase (TH), Dopamine Transporter (DAT), and VMAT2 from the control, MTPT, and lupeol-treated mice SNpc and striatum regions (n= 4) **(b–d)** Densitometry analysis of TH, DAT, and VMAT2 expression. **(e)** Double immunofluorescence staining for TH (TRITC-red) and Iba-1 (FITC-green), with DAPI nuclear staining (blue) counterstaining in the SNpc and striatum (n= 4). **(f, g)** Densitometry analysis of TH and Iba-1 immunofluorescence. β-Actin was used as a loading control. The cropped bands were quantified using Image software. Scale bar: 100µm. Data are presented as mean ± SEM. The hash symbol (#) denotes a significant difference from the control group, while the asterisk (*) indicates a significant difference from the MPTP-treated group. Statistical significance: ## p < 0.01; ###p < 0.001, ** p < 0.01; ***p < 0.001.

TH catalyzes the rate-limiting step in norepinephrine synthesis. A reduction in TH and norepinephrine levels leads to impaired dopamine production, contributing to PD (50, 51).To evaluate whether lupeol protects against MPTP-induced dopaminergic neuron loss in the SNpc and striatum, we performed Western blot analysis and immunofluorescence staining of brain sections. Our results indicate that MPTP-injected mice exhibited significantly lower TH expression in both the SNpc and striatum compared to controls. However, lupeol administration (50 mg/kg) significantly preserved TH expression relative to the MPTP-only group (**Figure 2A**). Additionally, we assessed DAT and VMAT2 levels by Western blot analysis, given that VMAT2 modulates MPTP toxicity in mice. MPTP treatment reduced DAT and VMAT2 expression in the SNpc and striatum, whereas lupeol administration significantly restored their levels (**Figures 2A–D**). To further validate these findings, we performed double immunofluorescence staining for TH and Iba-1 in the SNpc and striatum. MPTP-treated mice exhibited reduced TH expression and increased Iba-1 expression, indicative of microglial activation. Conversely, lupeol treatment preserved and enhanced TH expression while reducing Iba-1 expression in both the SNpc and striatum (**Figures 2E–G**). These results suggest that MPTP-induced microglial activation contributes to dopaminergic neuron loss, which is mitigated by lupeol treatment.

### 3.3 Lupeol reduces microglial activation and astrogliosis in the SNpc and striatum of MPTP-induced PD model mouse

**Figure 3:**
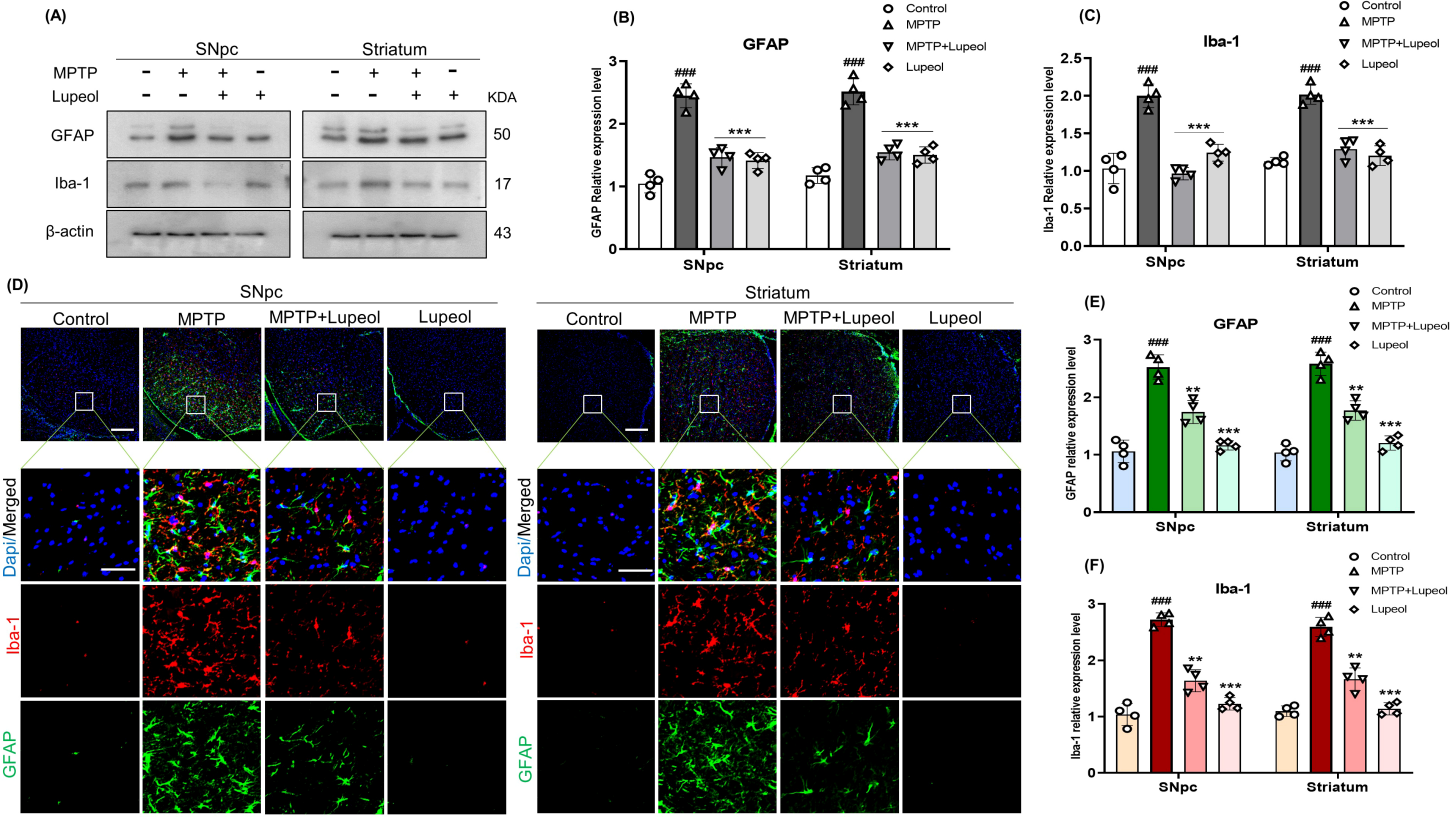
Lupeol suppresses glial cell activation in MPTP-induced PD model mice **(a)** Western blot analysis of GFAP and Iba-1 protein levels in the SNpc and striatum of control, MPTP-treated, and lupeol-treated mice (n= 4). **(b, c)** Densitometric analysis of GFAP and Iba-1 expression. **(d)** Double immunostaining of Iba-1 (TRITC-red) and GFAP (FITC-green), with DAPI nuclear staining (blue) in the SNpc and striatum (n= 4). **(e, f)** Quantification of GFAP and Iba-1 immunofluorescence intensity. β-Actin was used as a loading control. The cropped bands were quantified using Image software. Scale bar: 100µm; zoomed-in images: 40X magnification (scale bar 20µm). Data are presented as mean ± SEM. The hash (#) sign denotes a significant difference from the control group, while the asterisk (*) indicates a significant difference from the MPTP-treated group. Statistical significance: ###p < 0.001, ** p < 0.01; ***p < 0.001.

Reactive astrogliosis and microglial activation contribute to dopaminergic neuronal loss and neuroinflammation in PD (52). We assessed GFAP and Iba-1 expression, key astrocyte and microglial markers, in the SNpc and striatum of mice brains. Western blot analysis showed a significant increase in GFAP and Iba-1 expression following MPTP injection (30 mg/kg) compared to control mice. Lupeol administration (50 mg/kg) significantly reduced GFAP and Iba-1 levels compared to the MPTP-treated group (**Figures 3A–C**). Immunofluorescence analysis with GFAP and Iba-1 co-staining further confirmed these findings, demonstrating elevated expression in MPTP-treated mice, which was markedly reduced after lupeol administration (**Figures 3D–F**). These results suggest that lupeol mitigates MPTP-induced neuroinflammation by suppressing astrocyte and microglial activation.

### 3.4 Lupeol attenuates the MPTP-induced release of proinflammatory cytokines

**Figure 4:**
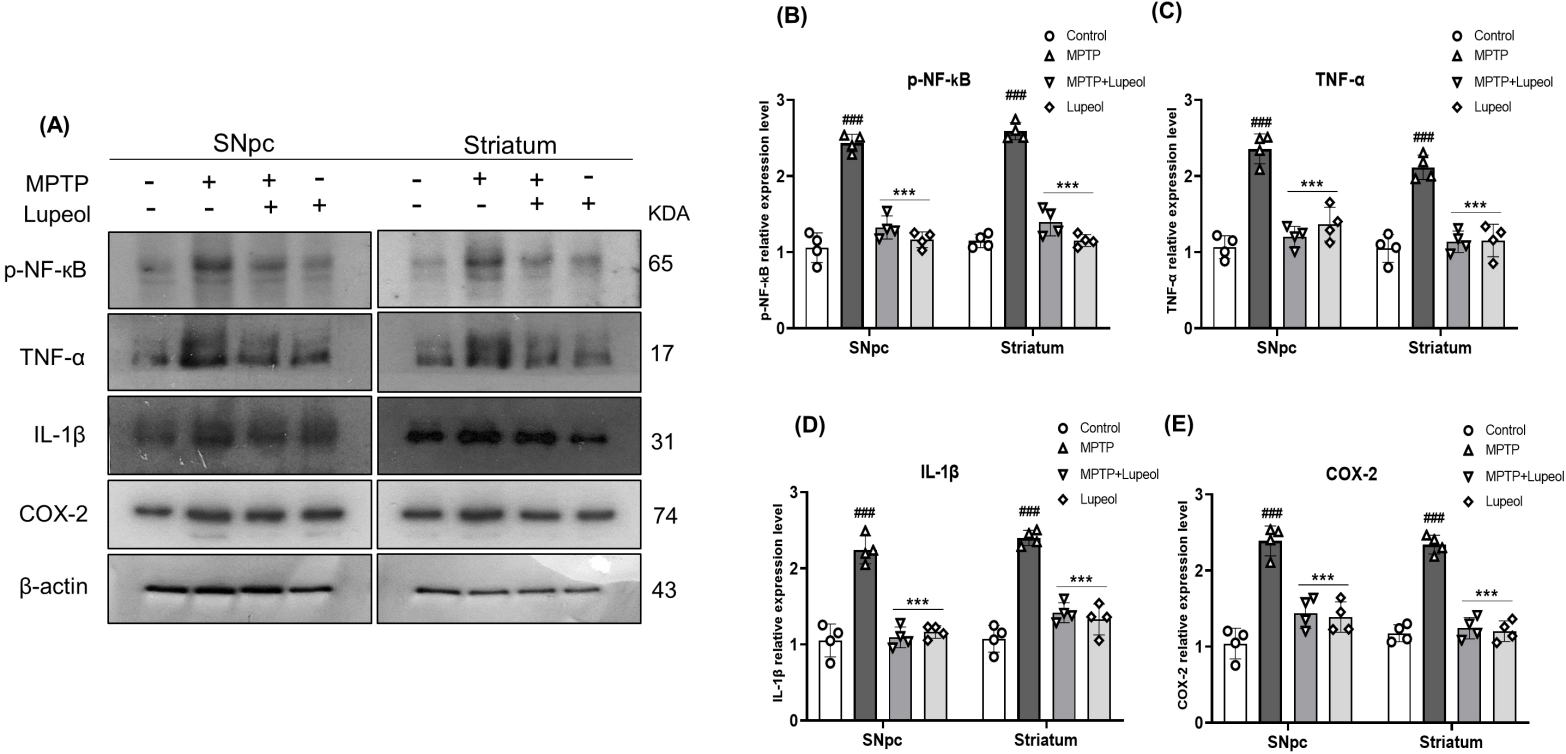
Lupeol inhibits the proinflammatory cytokine release mediated by MPTP. **(a)** Western blot analysis for detecting the protein level of p-NF-κB, TNF-α, IL-1β, and COX-2 of control, MTPT, and lupeol-treated mice (n= 4). **(b–e)** Densitometric analysis p-NF-κB, TNF-α, IL-1β, and COX-2. β-Actin was used as a loading control. The cropped bands were quantified using Image software. Data are presented as mean ± SEM. The hash (#) sign indicates a significant difference from the control group; the sign asterisk (*) indicates a significant difference from the MPTP-treated group. Statistical significance: ###p < 0.001, ***p < 0.001.

NF-κB activation induces the transcription of pro-inflammatory genes, leading to elevated levels of pro-inflammatory cytokines that contribute to neuroinflammation (53). To assess this, we analyzed NF-κB activation and the expression of pro-inflammatory cytokines, including IL-1β, TNF-α, and COX2, using western blot analysis. Our results revealed that MPTP treatment significantly activated NF-κB signaling, as evidenced by an increase in phosphorylated NF-κB (p-NF-κB) levels. This activation was associated with a marked upregulation of IL-1β, TNF-α, and COX2 in the SNpc and striatum compared to control mice, indicating a heightened inflammatory response in these brain regions. Importantly, administration of lupeol (50 mg/kg) effectively attenuated NF-κB activation, as reflected by a significant reduction in p-NF-κB expression. Furthermore, lupeol treatment markedly decreased the levels of IL-1β, TNF-α, and COX2, suggesting that its neuroprotective effects may be mediated, at least in part, by suppressing NF-κB-driven neuroinflammatory signaling (**Figure 4A–E**).

### 3.5 Lupeol exerts antioxidant effects in MPTP-treated mice

**Figure 5:**
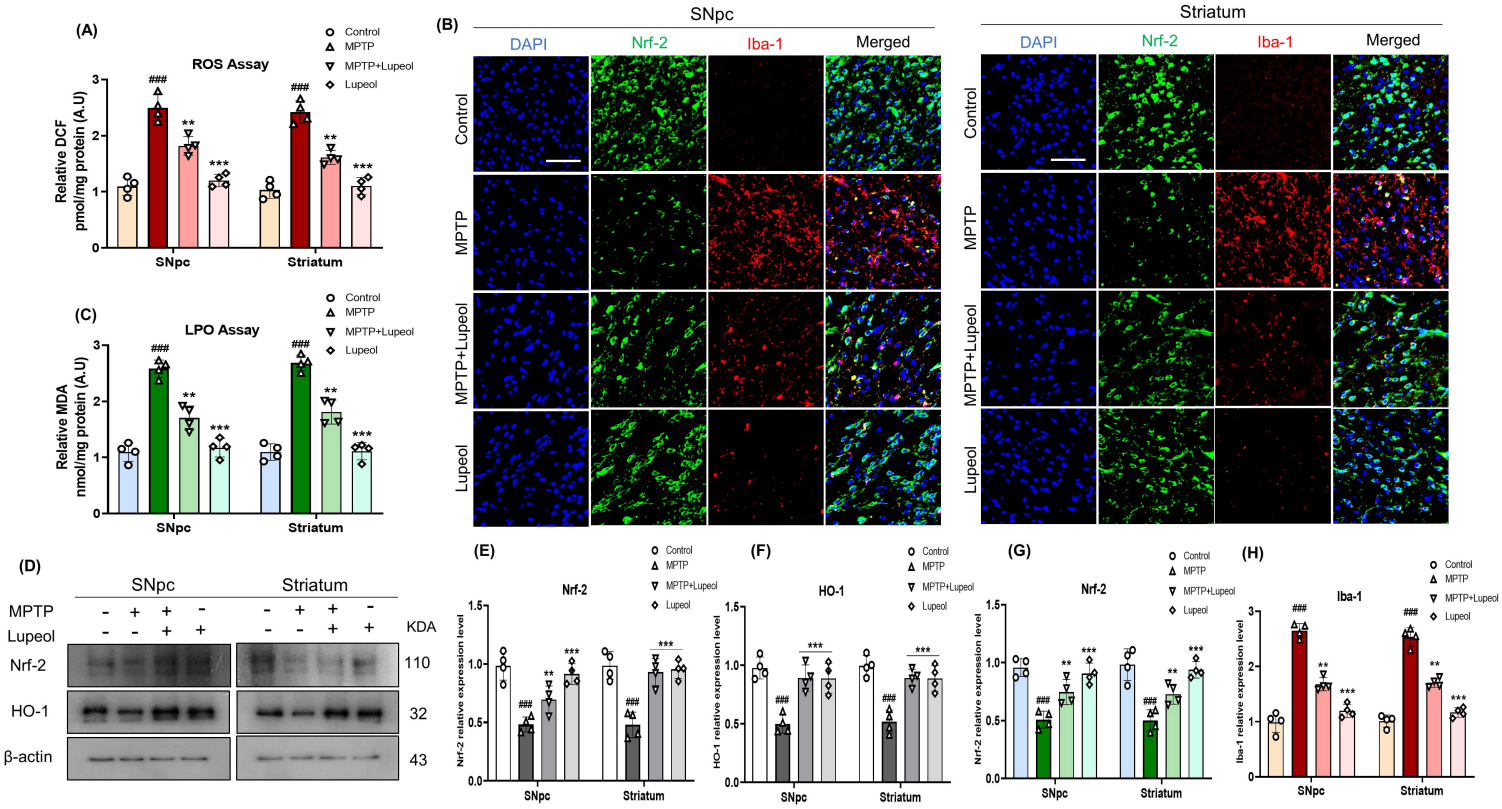
Lupeol exhibits antioxidant properties in MPTP-treated mice. **(a, c)** Quantitative analysis of ROS and LPO levels in all experimental groups. **(d)** Western blot analysis showing the protein expression of Nrf-2 and HO-1 in the SNpc and striatum of control, MPTP-treated, and lupeol-treated mice (n= 4). **(e, f)** Densitometric analysis of Nrf-2 and HO-1 expression. **(b)** Immunofluorescence staining of Nrf-2 (FITC-green) and Iba-1 (TRITC-red) with DAPI nuclear staining (blue) in the SNpc and striatum of experimental mice brain (n= 4). **(g, h)** Densitometric analysis of Nrf-2 and Iba-1 immunofluorescence. β-Actin was used as a loading control. The cropped bands were quantified using Image software. Scale bar: 20µm. Data are presented as mean ± SEM. The hash symbol (#) denotes a significant difference from the control group, while the asterisk (*) indicates a significant difference from the MPTP-treated group. Statistical significance: ###p < 0.001, ** p < 0.01; ***p < 0.001.

MPTP inhibits nicotinamide adenine dinucleotide dehydrogenase (NADH) in mitochondria, leading to excessive ROS generation which induces dopaminergic neuronal damage in the SNpc and striatum via lipid peroxidation (54, 55). To assess the protective effects of lupeol against MPTP-induced oxidative stress, we first measured ROS and LPO levels in all experimental groups. Our findings revealed a significant increase in ROS and LPO levels in the MPTP-treated group compared to controls. However, lupeol treatment effectively reduced these elevated ROS and LPO levels (**Figures 5A, C**). Next, we evaluated the expression of oxidative stress markers Nrf-2 and HO-1 using western blot analysis. MPTP treatment resulted in a marked reduction in Nrf-2 and HO-1 expression compared to control mice. Notably, lupeol administration significantly upregulated Nrf-2 and HO-1 expression in the SNpc and striatum (**Figures 5D–F**). To further confirm these findings, we performed immunofluorescence staining for Nrf-2 co-stained with Iba-1 in the SNpc and striatum. MPTP-treated mice exhibited decreased Nrf-2 expression and increased Iba-1 reactivity, indicating oxidative stress by microglial activation. In contrast, lupeol treatment preserved and enhanced Nrf-2 expression while reducing Iba-1 reactivity in both the SNpc and striatum (**Figures 5B, G, H**). These results suggest that lupeol mitigates MPTP-induced oxidative stress by reducing ROS and LPO levels while upregulating antioxidant defense mechanisms through Nrf-2 and HO-1 activation.

### 3.6 Lupeol treatment preserved NeuN-positive neuronal cells

**Figure 6:**
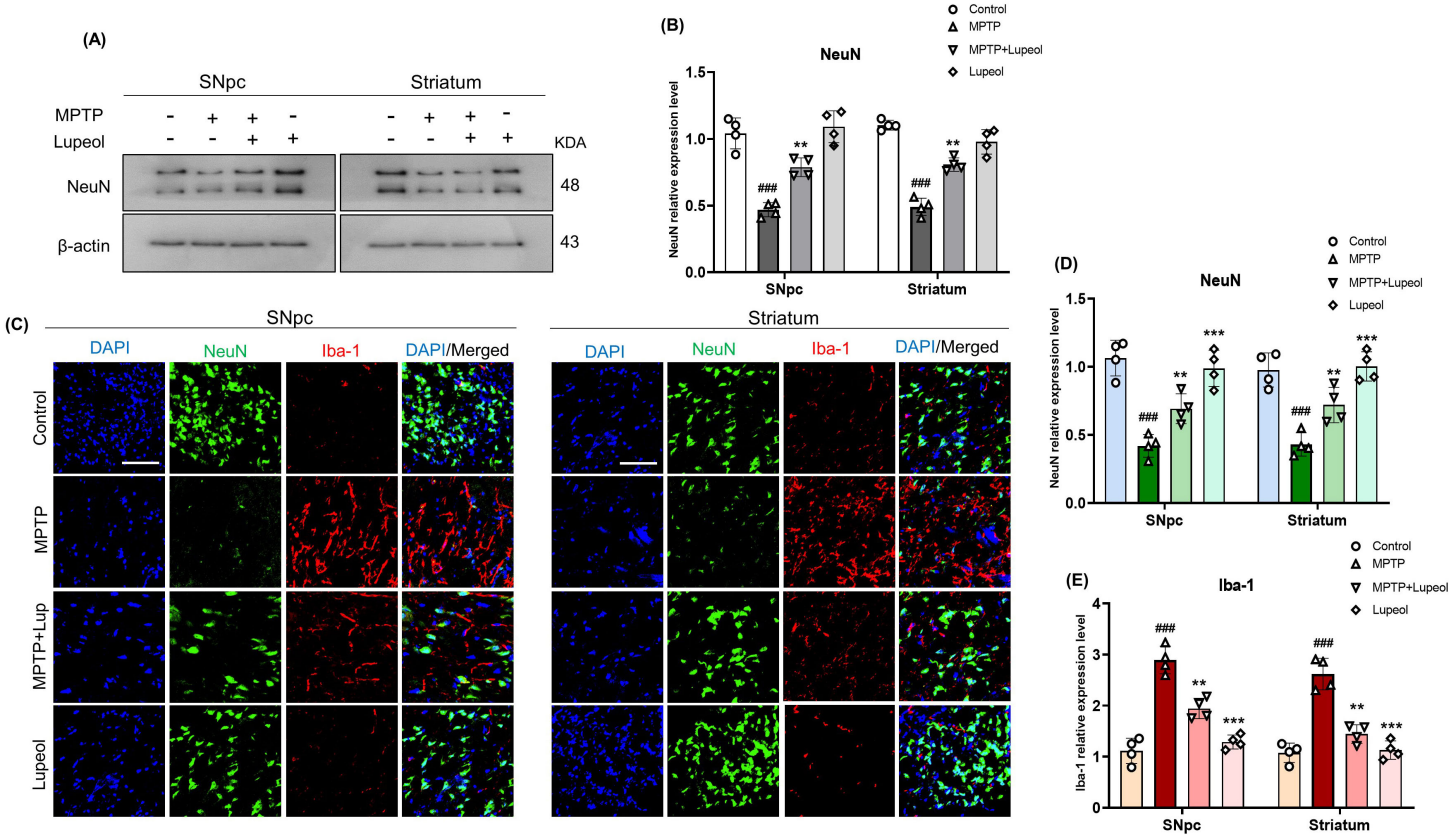
Lupeol Prevents MPTP-Induced Loss of NeuN-Positive Cells. **(a)** Western blot analysis showing NeuN protein levels in the SNpc and striatum regions of control, MPTP-treated, and lupeol-treated mice (n=4). **(b)** Densitometric analysis of NeuN expression. **(c)** Co-immunostaining of NeuN (FITC-green) and Iba-1 (TRITC-red), counterstained with DAPI nuclear staining (blue), in the SNpc and striatum regions of the mouse brain (n=4). **(d, e)** Densitometric analysis of NeuN and Iba-1 immunofluorescence. β-Actin was used as a loading control. The cropped bands were quantified using Image software. Scale bar: 50µm. Data are presented as mean ± SEM. The hash sign (#) indicates a significant difference from the control group, while the asterisk (*) indicates a significant difference from the MPTP-treated group. Statistical significance: ###p < 0.001, ** p < 0.01; ***p < 0.001.

Neuronal nuclear antigen (NeuN), a neuron-specific marker, is widely used to assess neuronal health and survival. MPTP exposure has been shown to alter NeuN expression in PD models (56). To assess whether microglial activation influences NeuN expression, Western blot analysis was performed. The results demonstrated a significant reduction in NeuN expression in the SNpc and striatum following MPTP administration, indicating neuronal loss (**Figures 6A, B**). Notably, lupeol treatment reversed this effect and upregulated NeuN expression in both the SNpc and striatum. Immunofluorescence analysis of NeuN co-stained with Iba-1 further confirmed these findings, demonstrating decreased NeuN expression and increased Iba-1 reactivity in MPTP-treated mice. Notably, lupeol treatment attenuated Iba-1 immunoreactivity and preserved NeuN-positive neuronal cells (**Figures 6C–E**).

### 3.7 Lupeol protects MPTP-induced apoptotic signaling in the SNpc and striatum of mouse brain

**Figure 7:**
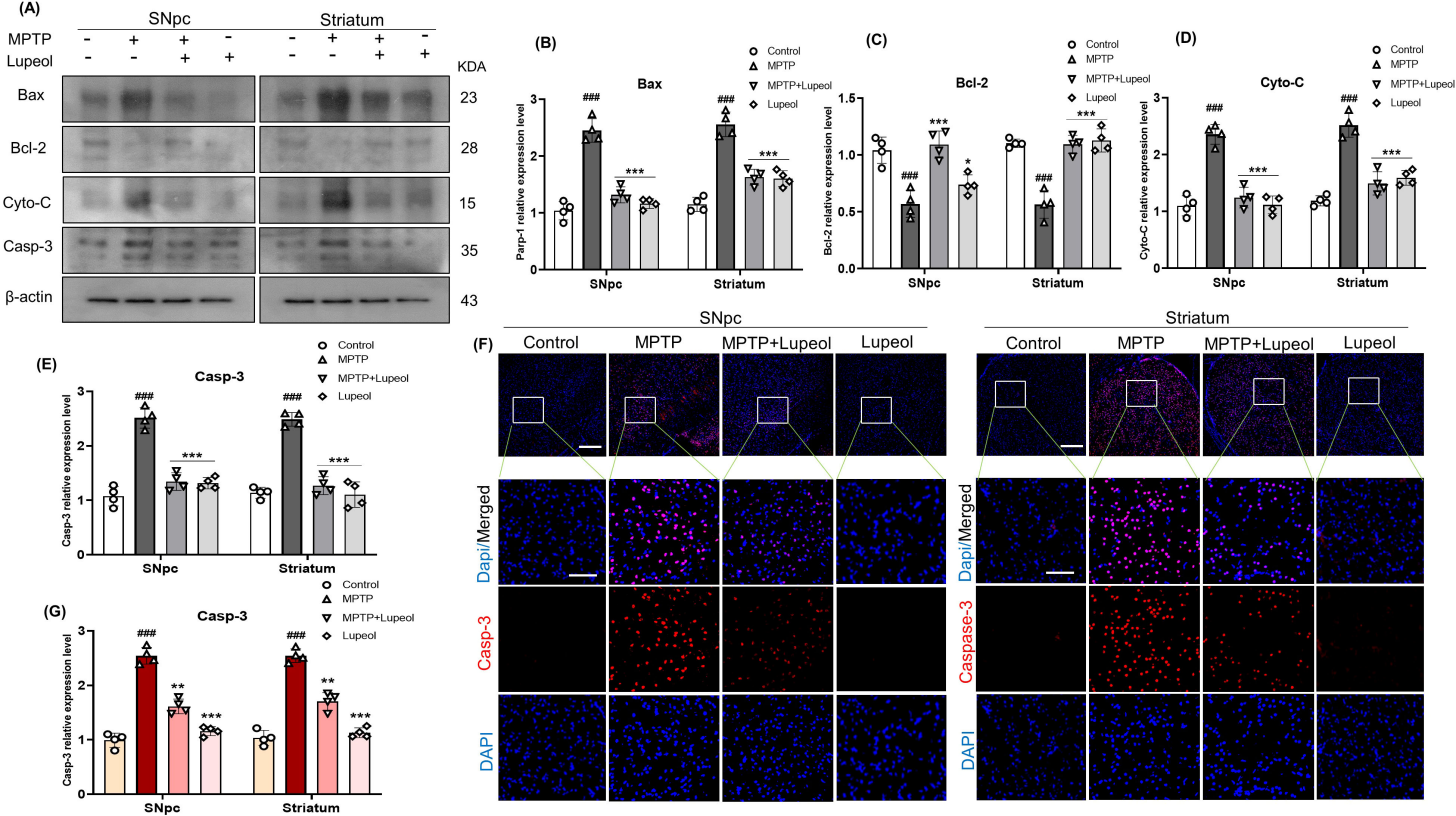
Lupeol prevents MPTP-induced apoptosis in Dopaminergic neurons. **(a)** Western blot analysis of Bax, Bcl-2, Cyto-C, and Casp-3 protein levels in the SNpc and striatum of control, MPTP-treated, and lupeol-treated mice (n= 4). **(b–e)** Densitometric analysis of Bax, Bcl-2, Cyto-C, and Casp-3 expression levels. **(f)** Densitometric analysis of Casp-3 immunofluorescence. **(g)** Immunofluorescence staining of Casp-3 (TRITC-red) with DAPI nuclear staining (blue) in the SNpc and striatum of mice brains (n= 4). β-Actin was used as a loading control. Cropped protein bands were quantified using ImageJ software. Scale bars: 100 µm (overview images) and 50 µm (zoomed-in images, 20X magnification). Data are presented as mean ± SEM. The hash symbol (#) denotes a significant difference from the control group, while the asterisk (*) denotes a significant difference from the MPTP-treated group. Statistical significance: ### p < 0.001, ** p < 0.01; *** p < 0.001.

Apoptotic signaling contributes to the progressive degeneration of dopaminergic neurons. Extensive research indicates that the dysregulation of pro-apoptotic and anti-apoptotic genes plays a critical role in DA neuronal cell death (57). In this study, MPTP treatment significantly upregulated the expression of apoptotic markers, including Bax, Cyto-C, and Casp-3, in the SNpc and striatum of the mouse brain. However, administration of lupeol (50 mg/kg) effectively downregulated the overexpression of Cyto-C, Parp-1, and Casp-3 in both brain regions (**Figures 7A–E**). Additionally, the expression of the anti-apoptotic marker Bcl-2, assessed via western blot analysis, was significantly reduced in MPTP-treated mice. Notably, lupeol administration restored Bcl-2 expression in the SNpc and striatum. To further validate these findings, we examined Casp-3 expression in the SNpc and striatum using immunofluorescence staining. Our results demonstrated a marked increase in Casp-3 expression in MPTP-treated mice, which was significantly reduced following lupeol treatment (**Figures 7F, G**).

## 4 Discussion

Parkinson’s disease causes motor performance deficiencies due to basal ganglia dysfunction, which includes neuroinflammation and dopaminergic cell loss (58). In our investigation, mice received subcutaneous injections of MPTP to mimic the lesions seen in PD patients. Previous research has shown that environmental toxicants can cause PD, and MPTP is a widely recognized chemical agent used to develop a PD animal model (59). The MPTP-exposed mouse model is the most frequent paradigm to examine the mechanism of PD-related cell death, as the dopaminergic neurotoxicity of MPTP causes dopamine depletion in both the SNpc and striatum, resulting in behavioral abnormalities (60). In mice, MPTP crosses the BBB and is metabolized to MPP+, an active toxic metabolite, particularly in astrocytes. Subsequently, MPP+ is then taken up by dopaminergic neurons, where it has damaging effects on mitochondria, resulting in oxidative stress and neuron death (61, 62). Several factors, including neuroinflammation, oxidative stress, Nrf2-dependent gene expression, α-synuclein aggregation, and mitochondrial dysfunction, are thought to impact the etiology of PD. This encourages a change away from “one-drug, one-target” pharmacology and towards studying medicines that can control the activity of several disease-relevant targets, potentially slowing or even halting nigrostriatal neurodegeneration in PD. Recently, there has been an increase in interest in employing natural compounds produced by plants to mitigate the progression of PD (63). In this study, we investigated the therapeutic efficacy of lupeol, a natural triterpenoid molecule, in a mouse model of Parkinson’s disease. The neuroprotective qualities of the triterpenoid Lupeol in a range of neurodegenerative and neuroinflammatory conditions have drawn more and more attention. Oral lupeol (50 mg/kg for two weeks) dramatically decreased oxidative stress (by activating the Nrf2 signaling pathway and inducing HO-1), inhibited glial activation and the production of inflammatory mediators, decreased the expression of Aβ and BACE-1, and enhanced memory in Alzheimer’s disease models (40). Lupeol therapy reduced ROS/lipid peroxidation, enhanced antioxidant proteins, inhibited apoptotic signaling, reduced microglial/astrocyte activation, and improved cognitive/behavioral results in traumatic brain damage models (39). Lupeol decreased neuronal damage, down-regulated iNOS, IL-1β, and TNF-α, and inhibited the p38-MAPK/JNK pathways in LPS-induced neuroinflammation models (64). The beneficial pharmacokinetic and pharmacodynamics characteristics of lupeol bolster its therapeutic promise in neurodegenerative studies. Because of its triterpenoid composition, lupeol has strong oral absorption, high lipid solubility, and effective blood–brain barrier (BBB) penetration, according to pharmacokinetic studies (65). It is mostly metabolized in the liver through phase I and II pathways, resulting in conjugated and hydroxylated metabolites that are eliminated by feces and bile. Lupeol functions as a strong antioxidant pharmacodynamically, boosting endogenous enzymes including SOD, CAT, and GPx and lowering lipid peroxidation. By inhibiting NF-κB, COX-2, TNF-α, IL-1β, and microglial activation, it also demonstrates anti-inflammatory properties. By modifying Bcl-2/Bax ratios, caspases, and mitochondrial stability, lupeol further exhibits anti-apoptotic activity (66). In our study, Lupeol reinstated motor coordination and reversed dopaminergic cell loss in the SN region, and the blunted neurotransmission toward the striatum. First, we discovered that MPTP treatment in mice caused motor impairments and decreased TH expression in the SNpc, which were mitigated by lupeol. Initially, we observed that MPTP administration caused motor deficits and decreased TH expression in the SNpc in mice, which were mitigated by lupeol. Second, MPTP boosted glial cell expression while simultaneously inducing oxidative stress, which was reduced by lupeol.

The neurotoxin MPTP can inflict severe damage on the nigrostriatal dopaminergic pathway in mice (67). When MPTP enters the body, it is converted into MPP+, an active neurotoxin that selectively enters norepinephrine (NE) and dopaminergic (DA) neurons through the norepinephrine transporter and dopamine transporter (13). MPP+ then forms a complex with neuromelanin in the axoplasm and is subsequently transported by VMAT-2 to be stored in synaptosomal vesicles (68). MPP+ continues to accumulate in synaptosomal vesicles until a threshold is surpassed, leading to the death of dopaminergic nigrostriatal neurons in the SNpc and striatum (10). Consequently, MPTP has also been shown to decrease the levels of TH in SNpc and striatum. Consistent with the previous studies, we observed the downregulation of TH, DAT, and VMAT2 in the SNpc and striatum of MPTP-treated mice. Fascinatingly, the lupeol treatment (50mg/kg) significantly reversed the effect of MPTP and increased the expression level of TH, DAT, and VMAT2 in both SNpc and striatum regions. Our study demonstrates that lupeol exhibits potent neurotrophic effects, protecting dopaminergic neurons from MPTP-induced degeneration.

Neuroinflammation intervened by glial cells and invading immune cells is a critical pathogenic characteristic of neurodegenerative diseases, including PD (69). Activation of glial cells contributes significantly to the beginning of nigrostriatal degeneration in PD patients and animals treated with MPTP. In addition, astrocytes are thought to be the principal targets of the offending substance. Upon activation, they exhibit rounded cell bodies and shrunken neurites that tend to cluster tightly together. Activated microglia displayed enlarged cell bodies and an amoeboid appearance, along with a reduced number of neurites. When glial cells become activated, they release proinflammatory cytokines and chemokines that contribute to neuronal death (61, 70). In this study, we found that MPTP boosted the expression of microglia and astrocytes in the SNpc and striatum areas of mouse brains. However, treatment with Lupeol reversed this activation. Furthermore, the transcription factor NF-kB plays a significant role in neuroinflammation, and its activation has been observed in postmortem brains of PD patients as well as and animal models (71, 72). Excessive cytotoxic substances, including nitric oxide, IL-1β, and TNFα, might result from prolonged or severe activation of NF-κB (73, 74). Therefore, we also measured the expression of proinflammatory molecules such as TNFα, COX2, and IL-1β, which are triggered by NF-κB activation. Following the activation of microglia and NF-κB by MPTP, we observed that the inflammatory cytokines IL-1β, TNF-α, and COX-2 expression were increased in the MPTP-treated mice group. However, the lupeol administration significantly reduced the production of these cytokines in the SNpc and striatum areas of the mouse brains.

Moreover, mitochondria, which are essential organelles required for cellular energy metabolism and redox homeostasis, play a role in apoptotic signaling pathways (27). Mitochondrial dysfunction and oxidative stress-induced apoptosis are critical components of PD etiology (75, 76). MPTP inhibits complex I of the mitochondrial electron transport chain (NADH-ubiquinone oxidoreductase), leading to decreased oxygen consumption, reduced ATP production, and disruptions in ion homeostasis, all of which contribute to increased formation of ROS (77). The excessive generation of ROS causes oxidative stress, which has been linked to the death of dopaminergic neurons in PD (78). In the current study, we found that MTPT administration enhanced the level of ROS and LPO in the SNpc and striatum areas of the mouse brains. Remarkably, the lupeol treatment reduced oxidative stress in dopaminergic neurons by inhibiting the formation of ROS and LPO in MPTP-treated dopaminergic neurons. Nrf2, a redox-sensitive master regulatory transcription factor, binds to the antioxidant response element in the promoter region of antioxidant enzymes like HO-1, thereby regulating their expression (79, 80). Activating Nrf2 enhances cellular redox homeostasis and generates cytoprotective responses; targeting Nrf2 signaling has recently shown therapeutic potential for PD (81). In our study, we observed the downregulation of Nrf2 and HO-1 in MPTP-treated mice, which were elevated by lupeol in the MPTP and lupeol co-treated mice group.

In addition, the deterioration of mitochondrial potential triggers the release of Cyto C into the cytoplasm, which activates caspase-3, and induces cell death (82). The Bcl-2 family plays a vital part in the mitochondrial apoptotic pathway, wherein Bcl-2 is an anti-apoptotic protein and Bax demonstrates pro-apoptotic activity (83). Bcl-2 suppresses caspase-3-dependent apoptosis by attaching to apoptosis-promoting Bax protein, therefore, Bcl-2/Bax ratio is a critical factor in determining whether cells undergo apoptotic (84, 85). Furthermore, Bax can form homodimers and trigger terminal caspases by altering mitochondrial activity after crossing through the mitochondrial membrane, releasing apoptosis-promoting substances into the cytoplasm (86). As a result, the Bcl-2/Bax ratio is extensively utilized to forecast the apoptosis of the cell (87). The Cyto C release from mitochondria to the cytosol activates Casp-3 and its downstream cell death pathways (88). Thus, Casp-3 activity is believed to be critical for committing to and carrying out neuronal death (89). Consistent with these results, we observed that MPTP caused overexpression of Cyto-C and Bax while downregulating the expression of Bcl-2 in SNpc and striatum of MPTP-treated mice, which might result in apoptosis. Our results demonstrated that lupeol greatly suppressed Cyto-C and Bax overexpression while decreasing Bcl-2 expression levels. Additionally, lupeol also inhibited the activation of Caspase-3 induced by MPTP. Taken together, our results imply that maintaining the balance between positive and negative regulators of apoptosis could be one of the mechanisms of lupeol’s neuroprotective activity.

Overall, Lupeol is usually considered safe and well-tolerated, with minimal acute and long-term toxicity, no significant alterations in hematological or biochemical indices, and no indication of organ toxicity at dosages that are often used. Only very high experimental doses cause mild side effects, such as temporary gastrointestinal distress or modest, reversible elevations in liver enzymes (90). Excellent tolerability is also shown when lupeol-rich plant extracts and nutraceutical formulations are used by humans. Although there is little data on drug interactions, *in vitro* and mechanistic investigations show that lupeol can alter cytochrome P450 enzymes, including CYP2C9 and CYP3A4, indicating possible interactions with medications that are metabolized by these routes. Combining lupeol with similar therapeutic drugs may result in additive or synergistic benefits because it also has anti-inflammatory and antioxidant properties (36). Although extensive human trials are still necessary to completely characterize safety margins and drug-interaction potential, present results generally show a robust safety profile.

## 5 Limitations and future perspectives

The acute MPTP mouse model employed in this study does not accurately reflect the long-term course of Parkinson’s disease. Because just one dose of lupeol was examined, dose-response effects could not be evaluated. While important indicators of apoptosis, oxidative stress, and neuroinflammation were looked at, upstream signaling pathways were not. Potential sex-related differences are unknown because only male mice were used. Furthermore, extended behavioral effects, long-term safety, and pharmacokinetic characteristics were not assessed.

In order to establish the best efficacy, future studies should test lupeol in hereditary and chronic Parkinson’s disease models and investigate various dosages and treatment durations. In-depth mechanistic research on proteostasis, inflammatory signaling, and mitochondrial control is required. Clinical significance will be ascertained with the aid of pharmacokinetic and bio distribution evaluations, including BBB penetration. Lupeol may have synergistic effects when combined with conventional PD treatments. To determine lupeol’s safety and therapeutic potential in people, extensive toxicity research and early-stage clinical trials are ultimately required.

## Data Availability

The original contributions presented in the study are included in the article/supplementary material. Further inquiries can be directed to the corresponding authors.

## References

[1] Hirsch L Jette N Frolkis A Steeves T Pringsheim T . The incidence of parkinson’s disease: A systematic review and meta-analysis. Neuroepidemiology. (2016) 46:292–300. doi: 10.1159/000445751, PMID: 27105081

[2] Moore DJ West AB Dawson VL Dawson TM . Molecular pathophysiology of Parkinson’s disease. Annu Rev Neurosci. (2005) 28:57–87. doi: 10.1146/annurev.neuro.28.061604.135718, PMID: 16022590

[3] Novikova L Garris BL Garris DR Lau YS . Early signs of neuronal apoptosis in the substantia nigra pars compacta of the progressive neurodegenerative mouse 1-methyl-4-phenyl-1,2,3,6-tetrahydropyridine/probenecid model of Parkinson’s disease. Neuroscience. (2006) 140:67–76. doi: 10.1016/j.neuroscience.2006.02.007, PMID: 16533572

[4] Luo SX Huang EJ . Dopaminergic neurons and brain reward pathways: from neurogenesis to circuit assembly. Am J Pathol. (2016) 186:478–88. doi: 10.1016/j.ajpath.2015.09.023, PMID: 26724386 PMC4816693

[5] Kravitz AV Freeze BS Parker PR Kay K Thwin MT Deisseroth K . Regulation of parkinsonian motor behaviours by optogenetic control of basal ganglia circuitry. Nature. (2010) 466:622–6. doi: 10.1038/nature09159, PMID: 20613723 PMC3552484

[6] Calabresi P Picconi B Tozzi A Ghiglieri V Di Filippo M . Direct and indirect pathways of basal ganglia: a critical reappraisal. Nat Neurosci. (2014) 17:1022–30. doi: 10.1038/nn.3743, PMID: 25065439

[7] Tritsch NX Sabatini BL . Dopaminergic modulation of synaptic transmission in cortex and striatum. Neuron. (2012) 76:33–50. doi: 10.1016/j.neuron.2012.09.023, PMID: 23040805 PMC4386589

[8] Ascherio A Schwarzschild MA . The epidemiology of Parkinson’s disease: risk factors and prevention. Lancet Neurol. (2016) 15:1257–72. doi: 10.1016/S1474-4422(16)30230-7, PMID: 27751556

[9] Dauer W Przedborski S . Parkinson’s disease: mechanisms and models. Neuron. (2003) 39:889–909. doi: 10.1016/S0896-6273(03)00568-3, PMID: 12971891

[10] Mustapha M Mat Taib CN . MPTP-induced mouse model of Parkinson’s disease: A promising direction of therapeutic strategies. Bosn J Basic Med Sci. (2021) 21:422–33. doi: 10.17305/bjbms.2020.5181, PMID: 33357211 PMC8292858

[11] Liu WW Wei SZ Huang GD Liu LB Gu C Shen Y . BMAL1 regulation of microglia-mediated neuroinflammation in MPTP-induced Parkinson’s disease mouse model. FASEB J. (2020) 34:6570–81. doi: 10.1096/fj.201901565RR, PMID: 32246801

[12] Schmidt N Ferger B . Neurochemical findings in the MPTP model of Parkinson’s disease. J Neural Transm (Vienna). (2001) 108:1263–82. doi: 10.1007/s007020100004, PMID: 11768626

[13] Meredith GE Rademacher DJ . MPTP mouse models of Parkinson’s disease: an update. J Parkinsons Dis. (2011) 1:19–33. doi: 10.3233/JPD-2011-11023, PMID: 23275799 PMC3530193

[14] Cao JJ Li KS Shen YQ . Activated immune cells in Parkinson’s disease. J Neuroimmune Pharmacol. (2011) 6:323–9. doi: 10.1007/s11481-011-9280-9, PMID: 21553347

[15] Litteljohn D Mangano E Clarke M Bobyn J Moloney K Hayley S . Inflammatory mechanisms of neurodegeneration in toxin-based models of Parkinson’s disease. Parkinsons Dis 2010. (2011) p:713517. doi: 10.4061/2011/713517, PMID: 21234362 PMC3018622

[16] Ferrari CC Pott Godoy MC Tarelli R Chertoff M Depino AM Pitossi FJ . Progressive neurodegeneration and motor disabilities induced by chronic expression of IL-1beta in the substantia nigra. Neurobiol Dis. (2006) 24:183–93. doi: 10.1016/j.nbd.2006.06.013, PMID: 16901708

[17] Gao HM Hong JS Zhang W Liu B . Distinct role for microglia in rotenone-induced degeneration of dopaminergic neurons. J Neurosci. (2002) 22:782–90. doi: 10.1523/JNEUROSCI.22-03-00782.2002, PMID: 11826108 PMC6758500

[18] Nicklas WJ Vyas I Heikkila RE . Inhibition of NADH-linked oxidation in brain mitochondria by 1-methyl-4-phenyl-pyridine, a metabolite of the neurotoxin, 1-methyl-4-phenyl-1,2,5,6-tetrahydropyridine. Life Sci. (1985) 36:2503–8. doi: 10.1016/0024-3205(85)90146-8, PMID: 2861548

[19] Mayer RA Kindt MV Heikkila RE . Prevention of the nigrostriatal toxicity of 1-methyl-4-phenyl-1,2,3,6-tetrahydropyridine by inhibitors of 3,4-dihydroxyphenylethylamine transport. J Neurochem. (1986) 47:1073–9. doi: 10.1111/j.1471-4159.1986.tb00722.x, PMID: 3489072

[20] Javitch JA D'Amato RJ Strittmatter SM Snyder SH . Parkinsonism-inducing neurotoxin, N-methyl-4-phenyl-1,2,3,6 -tetrahydropyridine: uptake of the metabolite N-methyl-4-phenylpyridine by dopamine neurons explains selective toxicity. Proc Natl Acad Sci U.S.A. (1985) 82:2173–7. doi: 10.1073/pnas.82.7.2173, PMID: 3872460 PMC397515

[21] Javitch JA Snyder SH . Uptake of MPP(+) by dopamine neurons explains selectivity of parkinsonism-inducing neurotoxin, MPTP. Eur J Pharmacol. (1984) 106:455–6. doi: 10.1016/0014-2999(84)90740-4, PMID: 6335692

[22] Del Zompo M Piccardi MP Ruiu S Quartu M Gessa GL Vaccari A . Selective MPP+ uptake into synaptic dopamine vesicles: possible involvement in MPTP neurotoxicity. Br J Pharmacol. (1993) 109:411–4. doi: 10.1111/j.1476-5381.1993.tb13584.x, PMID: 8102929 PMC2175677

[23] Mizuno Y Sone N Saitoh T . Effects of 1-methyl-4-phenyl-1,2,3,6-tetrahydropyridine and 1-methyl-4-phenylpyridinium ion on activities of the enzymes in the electron transport system in mouse brain. J Neurochem. (1987) 48:1787–93. doi: 10.1111/j.1471-4159.1987.tb05737.x, PMID: 3106573

[24] Rossetti ZL Sotgiu A Sharp DE Hadjiconstantinou M Neff NH . 1-Methyl-4-phenyl-1,2,3,6-tetrahydropyridine (MPTP) and free radicals *in vitro*. Biochem Pharmacol. (1988) 37:4573–4. doi: 10.1016/0006-2952(88)90674-0, PMID: 2849450

[25] Hasegawa E Takeshige K Oishi T Murai Y Minakami S . 1-Methyl-4-phenylpyridinium (MPP+) induces NADH-dependent superoxide formation and enhances NADH-dependent lipid peroxidation in bovine heart submitochondrial particles. Biochem Biophys Res Commun. (1990) 170:1049–55. doi: 10.1016/0006-291X(90)90498-C, PMID: 2167668

[26] Davey GP Clark JB . Threshold effects and control of oxidative phosphorylation in nonsynaptic rat brain mitochondria. J Neurochem. (1996) 66:1617–24. doi: 10.1046/j.1471-4159.1996.66041617.x, PMID: 8627318

[27] Bock FJ Tait SWG . Mitochondria as multifaceted regulators of cell death. Nat Rev Mol Cell Biol. (2020) 21:85–100. doi: 10.1038/s41580-019-0173-8, PMID: 31636403

[28] Vila M Jackson-Lewis V Vukosavic S Djaldetti R Liberatore G Offen D . Bax ablation prevents dopaminergic neurodegeneration in the 1-methyl- 4-phenyl-1,2,3,6-tetrahydropyridine mouse model of Parkinson’s disease. Proc Natl Acad Sci U.S.A. (2001) 98:2837–42., PMID: 11226327 10.1073/pnas.051633998 PMC30226

[29] Hassouna I Wickert H Zimmermann M Gillardon F . Increase in bax expression in substantia nigra following 1-methyl-4-phenyl-1,2,3,6-tetrahydropyridine (MPTP) treatment of mice. Neurosci Lett. (1996) 204:85–8. doi: 10.1016/0304-3940(96)12323-5, PMID: 8929984

[30] Correction for Perni . A natural product inhibits the initiation of alpha-synuclein aggregation and suppresses its toxicity. Proc Natl Acad Sci U.S.A. (2017) 114:E2543. 28096355 10.1073/pnas.1610586114 PMC5307473

[31] Sun CP Zhou JJ Yu ZL Huo XK Zhang J Morisseau C . Kurarinone alleviated Parkinson’s disease via stabilization of epoxyeicosatrienoic acids in animal model. Proc Natl Acad Sci U.S.A. (2022) 119. doi: 10.1073/pnas.2118818119, PMID: 35217618 PMC8892522

[32] Fox SH Katzenschlager R Lim SY Barton B De Bie RM Seppi K . International Parkinson and movement disorder society evidence-based medicine review: update on treatments for the motor symptoms of Parkinson’s disease. Mov. Disord. (2018) 33:1248–66. doi: 10.1002/mds.27372, PMID: 29570866

[33] Marino BL de Souza LR Sousa KP Ferreira JV Padilha EC da Silva CH . Parkinson’s disease: a review from pathophysiology to treatment. Med. Chem. (2020) 20:754–67. doi: 10.2174/1389557519666191104110908 31686637

[34] Saleem M . Lupeol, a novel anti-inflammatory and anti-cancer dietary triterpene. Cancer Lett. (2009) 285:109–15. doi: 10.1016/j.canlet.2009.04.033, PMID: 19464787 PMC2764818

[35] Siddique HR Saleem M . Beneficial health effects of lupeol triterpene: a review of preclinical studies. Life Sci. (2011) 88:285–93. doi: 10.1016/j.lfs.2010.11.020, PMID: 21118697

[36] Liu K Zhang X Xie L Deng M Chen H Song J . Lupeol and its derivatives as anticancer and anti-inflammatory agents: Molecular mechanisms and therapeutic efficacy. Pharmacol Res. (2021) 164:105373. doi: 10.1016/j.phrs.2020.105373, PMID: 33316380

[37] Ahmad R Khan A Lee HJ Ur Rehman I Khan I Alam SI . Lupeol, a plant-derived triterpenoid, protects mice brains against abeta-induced oxidative stress and neurodegeneration. Biomedicines. (2020) 8. doi: 10.3390/biomedicines8100380, PMID: 32993092 PMC7601269

[38] Badshah H Ali T Shafiq-ur R Faiz-ul A Ullah F Kim TH . Protective Effect of Lupeol Against Lipopolysaccharide-Induced Neuroinflammation via the p38/c-Jun N-Terminal Kinase Pathway in the Adult Mouse Brain. J Neuroimmune Pharmacol. (2016) 11:48–60. doi: 10.1007/s11481-015-9623-z, PMID: 26139594

[39] Ahmad R Khan A Rehman IU Lee HJ Khan I Kim MO . Lupeol treatment attenuates activation of glial cells and oxidative-stress-mediated neuropathology in mouse model of traumatic brain injury. Int J Mol Sci. (2022) 23. doi: 10.3390/ijms23116086, PMID: 35682768 PMC9181489

[40] Ahmad R Khan A Lee HJ Ur Rehman I Khan I Alam SI . Lupeol, a plant-derived triterpenoid, protects mice brains against Aβ-induced oxidative stress and neurodegeneration. Biomedicines. (2020) 8:380. doi: 10.3390/biomedicines8100380, PMID: 32993092 PMC7601269

[41] He XJ Yamauchi H Uetsuka K Nakayama HJN . Neurotoxicity of MPTP to migrating neuroblasts: studies in acute and subacute mouse models of Parkinson’s disease. Neurotoxicology. (2008) 29:413–20. doi: 10.1016/j.neuro.2008.02.007, PMID: 18387672

[42] Qi X-H Chen P Wang Y-J Zhou Z-P Liu X-C Fang H . Increased cysteinyl-tRNA synthetase drives neuroinflammation in Alzheimer’s disease. Transl. Neurodegener. (2024) 13:3. doi: 10.1186/s40035-023-00394-6, PMID: 38191451 PMC10773087

[43] Qin X Wang S Huang J Hu B Yang X Liang L . Rhein alleviates MPTP-induced Parkinson’s disease by suppressing neuroinflammation via MAPK/IκB pathway. Front. Neurosci. (2024) 18:1396345. doi: 10.3389/fnins.2024.1396345, PMID: 38933815 PMC11202316

[44] Leem Y-H Park J-S Park J-E Kim D-Y Kim H-SJSr . Neurogenic effects of rotarod walking exercise in subventricular zone, subgranular zone, and substantia nigra in MPTP-induced Parkinson’s disease mice. Sci. Rep. (2022) 12:10544. doi: 10.1038/s41598-022-14823-5, PMID: 35732806 PMC9217938

[45] Zhou Y Zhao WJ Quan W Qiao CM Cui C Hong H . Dynamic changes of activated AHR in microglia and astrocytes in the substantia nigra-striatum system in an MPTP-induced Parkinson’s disease mouse model. Brain Res Bull. (2021) 176:174–83. doi: 10.1016/j.brainresbull.2021.08.013, PMID: 34478811

[46] Sun L Wang H Yu S Zhang L Jiang J Zhou QJI . Herceptin induces ferroptosis and mitochondrial dysfunction in H9c2 cells. (2022) 49:1–8., PMID: 34935058 10.3892/ijmm.2021.5072 PMC8711589

[47] Rehman SU Shah SA Ali T Chung JI Kim MO . Anthocyanins reversed D-galactose-induced oxidative stress and neuroinflammation mediated cognitive impairment in adult rats. Mol Neurobiol. (2017) 54:255–71. doi: 10.1007/s12035-015-9604-5, PMID: 26738855

[48] Ciani C Pistorio G Mearelli M Falcone CJS . Immunofluorescence protocol for localizing protein targets in brain tissue from diverse model and non-model mammals. STAR Protoc. (2023) 4:102482. doi: 10.1016/j.xpro.2023.102482, PMID: 37561635 PMC10432796

[49] Samson AL Ju L Ah Kim H Zhang SR Lee JA Sturgeon SA . MouseMove: an open source program for semi-automated analysis of movement and cognitive testing in rodents. Sci Rep. (2015) 5:16171. doi: 10.1038/srep16171, PMID: 26530459 PMC4632026

[50] DiFrancisco-Donoghue J Rabin E Lamberg EM Werner WG . Effects of tyrosine on parkinson’s disease: A randomized, double-blind, placebo-controlled trial. Mov Disord Clin Pract. (2014) 1:348–53., PMID: 30363894 10.1002/mdc3.12082 PMC6183247

[51] Zhu Y Zhang J Zeng Y . Overview of tyrosine hydroxylase in Parkinson’s disease. CNS Neurol Disord Drug Targets. (2012) 11:350–8. doi: 10.2174/187152712800792901, PMID: 22483316

[52] Teismann P Schulz JB . Cellular pathology of Parkinson’s disease: astrocytes, microglia and inflammation. Cell Tissue Res. (2004) 318:149–61. doi: 10.1007/s00441-004-0944-0, PMID: 15338271

[53] Aoki E Yano R Yokoyama H Kato H Araki T . Role of nuclear transcription factor kappa B (NF-kappaB) for MPTP (1-methyl-4-phenyl-1,2,3,6-tetrahyropyridine)-induced apoptosis in nigral neurons of mice. Exp Mol Pathol. (2009) 86:57–64. doi: 10.1016/j.yexmp.2008.10.004, PMID: 19027004

[54] Andreassen OA Ferrante RJ Dedeoglu A Albers DW Klivenyi P Carlson EJ . Mice with a partial deficiency of manganese superoxide dismutase show increased vulnerability to the mitochondrial toxins malonate, 3-nitropropionic acid, and MPTP. Exp Neurol. (2001) 167:189–95. doi: 10.1006/exnr.2000.7525, PMID: 11161607

[55] Chun HS Gibson GE DeGiorgio LA Zhang H Kidd VJ Son JH . Dopaminergic cell death induced by MPP(+), oxidant and specific neurotoxicants shares the common molecular mechanism. J Neurochem. (2001) 76:1010–21., PMID: 11181820 10.1046/j.1471-4159.2001.00096.x

[56] Stayte S Rentsch P Troscher AR Bamberger M Li KM Vissel B . Activin A inhibits MPTP and LPS-induced increases in inflammatory cell populations and loss of dopamine neurons in the mouse midbrain *in vivo*. PloS One. (2017) 12:e0167211. doi: 10.1371/journal.pone.0167211, PMID: 28121982 PMC5266209

[57] Erekat NS . Apoptosis and its Role in Parkinson’s Disease. In: Stoker TB Greenland JC , editors. Parkinson’s Disease: Pathogenesis and Clinical Aspects. Brisbane (AU: Codon Publications (2018).

[58] Onate M Catenaccio A Salvadores N Saquel C Martinez A Moreno-Gonzalez I . The necroptosis machinery mediates axonal degeneration in a model of Parkinson disease. Cell Death Differ. (2020) 27:1169–85. doi: 10.1038/s41418-019-0408-4, PMID: 31591470 PMC7205895

[59] Langston JW Ballard P Tetrud JW Irwin I . Chronic Parkinsonism in humans due to a product of meperidine-analog synthesis. Science. (1983) 219:979–80. doi: 10.1126/science.6823561, PMID: 6823561

[60] Wang Q Ren N Cai Z Lin Q Wang Z Zhang Q . Paraquat and MPTP induce neurodegeneration and alteration in the expression profile of microRNAs: the role of transcription factor Nrf2. NPJ Parkinsons Dis. (2017) 3:31. doi: 10.1038/s41531-017-0033-1, PMID: 29071302 PMC5651826

[61] Zeng XS Geng WS Jia JJ . Neurotoxin-induced animal models of parkinson disease: pathogenic mechanism and assessment. ASN Neuro. (2018) 10:1759091418777438. doi: 10.1177/1759091418777438, PMID: 29809058 PMC5977437

[62] Abushouk AI Negida A Ahmed H Abdel-Daim MM . Neuroprotective mechanisms of plant extracts against MPTP induced neurotoxicity: Future applications in Parkinson’s disease. BioMed Pharmacother. (2017) 85:635–45. doi: 10.1016/j.biopha.2016.11.074, PMID: 27890431

[63] Kim SW Lee JH Kim B Yang G Kim JU . Natural products as the potential to improve alzheimer’s and parkinson’s disease. Int J Mol Sci. (2023) 24. doi: 10.3390/ijms24108827, PMID: 37240173 PMC10218422

[64] Choe K Park JS Park HY Tahir M Park TJ Kim MOJF . Lupeol protect against LPS-induced neuroinflammation and amyloid beta in adult mouse hippocampus. Int. J. Mol. Sci. (2024) 11:1414696. doi: 10.3389/fnut.2024.1414696, PMID: 39050141 PMC11266137

[65] Sohag AAM Hossain MT Rahaman MA Rahman P Hasan MS Das RC . Molecular pharmacology and therapeutic advances of the pentacyclic triterpene lupeol. Front. Nutr. (2022) 99:154012. doi: 10.1016/j.phymed.2022.154012, PMID: 35286936

[66] Fatma H Jameel M Akhtar K Ansari MA Siddique HRJL . Implication of Lupeol in compensating Sorafenib-induced perturbations of redox homeostasis: a preclinical study in mouse model. Phytomedicine. (2023) 322:121647. doi: 10.1016/j.lfs.2023.121647, PMID: 37011877

[67] Heikkila RE Hess A Duvoisin RC . Dopaminergic neurotoxicity of 1-methyl-4-phenyl-1,2,5,6-tetrahydropyridine in mice. Science. (1984) 224:1451–3. doi: 10.1126/science.6610213, PMID: 6610213

[68] Gainetdinov RR Fumagalli F Wang YM Jones SR Levey AI Miller GW . Increased MPTP neurotoxicity in vesicular monoamine transporter 2 heterozygote knockout mice. J Neurochem. (1998) 70:1973–8. doi: 10.1046/j.1471-4159.1998.70051973.x, PMID: 9572281

[69] Wang Q Liu Y Zhou J . Neuroinflammation in Parkinson’s disease and its potential as therapeutic target. Transl Neurodegener. (2015) 4:19. doi: 10.1186/s40035-015-0042-0, PMID: 26464797 PMC4603346

[70] Nam J Richie CT Harvey BK Voutilainen MH . Delivery of CDNF by AAV-mediated gene transfer protects dopamine neurons and regulates ER stress and inflammation in an acute MPTP mouse model of Parkinson’s disease. Sci Rep. (2024) 14:16487. doi: 10.1038/s41598-024-65735-5, PMID: 39019902 PMC11254911

[71] Hunot S Brugg B Ricard D Michel PP Muriel MP Ruberg M . Nuclear translocation of NF-kappaB is increased in dopaminergic neurons of patients with parkinson disease. Proc Natl Acad Sci U.S.A. (1997) 94:7531–6., PMID: 9207126 10.1073/pnas.94.14.7531 PMC23856

[72] Ghosh A Roy A Liu X Kordower JH Mufson EJ Hartley DM . Selective inhibition of NF-kappaB activation prevents dopaminergic neuronal loss in a mouse model of Parkinson’s disease. Proc Natl Acad Sci U.S.A. (2007) 104:18754–9., PMID: 18000063 10.1073/pnas.0704908104 PMC2141849

[73] Block ML Zecca L Hong JS . Microglia-mediated neurotoxicity: uncovering the molecular mechanisms. Nat Rev Neurosci. (2007) 8:57–69. doi: 10.1038/nrn2038, PMID: 17180163

[74] Hirsch EC Hunot S . Neuroinflammation in Parkinson’s disease: a target for neuroprotection? Lancet Neurol. (2009) 8:382–97. 10.1016/S1474-4422(09)70062-6 19296921

[75] Pickrell AM Youle RJ . The roles of PINK1, parkin, and mitochondrial fidelity in Parkinson’s disease. Neuron. (2015) 85:257–73. doi: 10.1016/j.neuron.2014.12.007, PMID: 25611507 PMC4764997

[76] Mani S Sevanan M Krishnamoorthy A Sekar S . A systematic review of molecular approaches that link mitochondrial dysfunction and neuroinflammation in Parkinson’s disease. Neurol Sci. (2021) 42:4459–69. doi: 10.1007/s10072-021-05551-1, PMID: 34480241

[77] Przedborski S Jackson-Lewis V Djaldetti R Liberatore G Vila M Vukosavic S . The parkinsonian toxin MPTP: action and mechanism. Restor Neurol Neurosci. (2000) 16:135–42. doi: 10.3233/RNN-2000-00132 12671216

[78] Subramaniam SR Chesselet MF . Mitochondrial dysfunction and oxidative stress in Parkinson’s disease. Prog Neurobiol. (2013) 106-107:17–32. doi: 10.1016/j.pneurobio.2013.04.004, PMID: 23643800 PMC3742021

[79] Itoh K Wakabayashi N Katoh Y Ishii T O'Connor T Yamamoto M . Keap1 regulates both cytoplasmic-nuclear shuttling and degradation of Nrf2 in response to electrophiles. Genes Cells. (2003) 8:379–91. doi: 10.1046/j.1365-2443.2003.00640.x, PMID: 12653965

[80] Lee JM Calkins MJ Chan K Kan YW Johnson JA . Identification of the NF-E2-related factor-2-dependent genes conferring protection against oxidative stress in primary cortical astrocytes using oligonucleotide microarray analysis. J Biol Chem. (2003) 278:12029–38. doi: 10.1074/jbc.M211558200, PMID: 12556532

[81] Saha S Buttari B Panieri E Profumo E Saso L . An overview of nrf2 signaling pathway and its role in inflammation. Molecules. (2020) 25. doi: 10.3390/molecules25225474, PMID: 33238435 PMC7700122

[82] Jovanovic-Tucovic M Harhaji-Trajkovic L Dulovic M Tovilovic-Kovacevic G Zogovic N Jeremic M . AMP-activated protein kinase inhibits MPP+-induced oxidative stress and apoptotic death of SH-SY5Y cells through sequential stimulation of Akt and autophagy. Eur J Pharmacol. (2019) 863:172677. doi: 10.1016/j.ejphar.2019.172677, PMID: 31542478

[83] Gross A McDonnell JM Korsmeyer SJ . BCL-2 family members and the mitochondria in apoptosis. Genes Dev. (1999) 13:1899–911. doi: 10.1101/gad.13.15.1899, PMID: 10444588

[84] Kerr JF Wyllie AH Currie AR . Apoptosis: a basic biological phenomenon with wide-ranging implications in tissue kinetics. Br J Cancer. (1972) 26:239–57. doi: 10.1038/bjc.1972.33, PMID: 4561027 PMC2008650

[85] Reed JC . Bcl-2 and the regulation of programmed cell death. J Cell Biol. (1994) 124:1–6. doi: 10.1083/jcb.124.1.1, PMID: 8294493 PMC2119888

[86] Adams JM Cory S . The Bcl-2 protein family: arbiters of cell survival. Science. (1998) 281:1322–6. doi: 10.1126/science.281.5381.1322, PMID: 9735050

[87] Raisova M Hossini AM Eberle J Riebeling C Wieder T Sturm I . The Bax/Bcl-2 ratio determines the susceptibility of human melanoma cells to CD95/Fas-mediated apoptosis. J Invest Dermatol. (2001) 117:333–40. doi: 10.1046/j.0022-202x.2001.01409.x, PMID: 11511312

[88] Skulachev VP . Cytochrome c in the apoptotic and antioxidant cascades. FEBS Lett. (1998) 423:275–80. doi: 10.1016/S0014-5793(98)00061-1, PMID: 9515723

[89] Harada J Sugimoto M . Activation of caspase-3 in beta-amyloid-induced apoptosis of cultured rat cortical neurons. Brain Res. (1999) 842:311–23. doi: 10.1016/S0006-8993(99)01808-9, PMID: 10526127

[90] Luo X Li J Cen Z Feng G Hong M Huang L . Exploring the therapeutic potential of lupeol: A review of its mechanisms, clinical applications, and advances in bioavailability enhancement. Brain Res. (2025) 196:115193. doi: 10.1016/j.fct.2024.115193, PMID: 39662867

